# Current and time‐lagged effects of climate on innate immunity in two sympatric snake species

**DOI:** 10.1002/ece3.7273

**Published:** 2021-02-16

**Authors:** Lucia L. Combrink, Anne M. Bronikowski, David A. W. Miller, Amanda M. Sparkman

**Affiliations:** ^1^ Westmont College Santa Barbara CA USA; ^2^ Iowa State University Ames IA USA; ^3^ Pennsylvania State University State College PA USA

**Keywords:** drought, garter snakes, innate immunity, precipitation, sympatry, temperature

## Abstract

1. Changing environments result in alterations at all levels of biological organization, from genetics to physiology to demography. The increasing frequency of droughts worldwide is associated with higher temperatures and reduced precipitation that can impact population persistence via effects on individual immune function and survival.
2. We examined the effects of annual climate variation on immunity in two sympatric species of garter snakes from four populations in California over a seven‐year period that included the record‐breaking drought.
3. We examined three indices of innate immunity: bactericidal competence (BC), natural antibodies (NABs), and complement‐mediated lysis (CL).
4. Precipitation was the only climatic variable explaining variation in immune function: spring precipitation of the current year was positively correlated to *Thamnophis sirtalis* BC and NABs, whereas spring precipitation of the previous year was positively correlated to *T. elegans* BC and NABs. This suggests that *T. elegans* experiences a physiological time‐lag in response to reduced precipitation, which may reflect lack of capital for investment in immunity in the year following a dry year.
5. In general, our findings demonstrate compelling evidence that climate can influence wild populations through effects on physiological processes, suggesting that physiological indices such as these may offer valuable opportunities for monitoring the effects of climate.

## Graphical abstract

We present seven years of data on four populations of two sympatric species of garter snake to test for effects of climate on three indices of innate immunity. We found that precipitation is significantly related to immunity in both species, with an important difference: precipitation of the previous year is related to immunity for one species, whereas precipitation of the current year is most important for the other. Thus, this study is the first to show a time‐lagged effect of climate on immune function in the wild and constitutes a powerful example of interspecific differences in response to environmental change even in closely related sympatric species

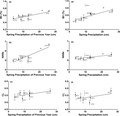

## 1 INTRODUCTION

Increasing global temperatures in recent years have led to rapidly changing environments around the world, often with profound effect on species and their survival (see Thomas et al., 2004; Walther et al., 2002). As many as 15%–37% of species are projected to be extinct by 2050, with many already experiencing difficulties from climate‐induced stress, resulting in range constriction and alterations in reproductive phenology (e.g., Barnosky et al., 2011; Chen et al., 2011; Janzen et al., 2018; Miller et al., 2018; Stuart et al., 2004; Thomas et al., 2004; Urban, 2015). These changes call for intensive research on diverse species and ecosystems to deepen our understanding of how organisms respond to rising temperatures, changes in precipitation, resource scarcity, and other rapid shifts in environmental variables affecting different levels of biological organization, from physiology to demography to community and ecosystem function.

Climate change has been shown to increase the intensity, duration, and frequency of droughts worldwide (Allen et al., 2010; Kelley et al., 2015). For instance, the Syrian drought from 2007 to 2010 occurred due to human‐induced global climate change and led to mass urbanization and civil war (Kelley et al., 2015). Desertification in the African Sahel has also been connected to climate change and that region is projected to become more arid in the future (Benjaminsen & Hiernaux, 2019; Huang et al., 2016). Similarly, the recent and record‐breaking drought of 2012–2015 in California, called the most severe drought in that region in the last century, has been widely attributed to climate change (Griffin & Anchukaitis, 2014, p. 9,017; Mann & Gleick, 2015; Swain et al., 2014; Bales et al., 2018; Sumargo & Cayan, 2018; Asner et al., 2016).

A handful of studies to date have examined the impact of drought on mortality and survival in resident species. Prugh et al. (2018) examined 423 species for several years before and during the drought and classified 25% as ecological “losers” in drought conditions and only 4% as “winners.” Previous research on endangered anurans in California has found that droughts lead to rapid species decline and habitat loss (Drost & Fellers, 1996; Fellers & Drost, 1993). Reptiles and amphibians are especially vulnerable to climate change, particularly in areas where extreme temperature and drought are predicted to become more severe and frequent (Corn, 2005; Thomas et al., 2004; Walls et al., 2013; Walther et al., 2002). These taxa are ectothermic and rely on ambient temperatures to regulate physiological functions. In fact, rising global temperatures already threaten the survival of tropical lizards that often function within a relatively low and narrow range of optimal body temperature (Huey et al., 2009). Furthermore, temperature and precipitation help regulate the breeding cycle of reptiles and amphibians (Araújo et al., 2006; Zani & Stein, 2018). The western side‐blotched lizard (*Uta stansburiana*), for example, experiences delays in breeding due to water deprivation during periods of drought (Zani & Stein, 2018). Similarly, a field study found female rattlesnakes experiencing drought did not lay eggs, whereas experimentally hydrated females did (Capehart et al., 2016). Reptiles and amphibians are also relatively immobile over long distances, unlike birds or some mammals, such that environmental change may pose a particularly significant threat.

Higher temperatures and reduced precipitation not only affect single species, but many species within a community's food web (Carnicer et al., 2011; Miller et al., 2018; Rosenblatt & Schmitz, 2016). Thus, drought may both directly affect a population's vital rates and also exert indirect effects through drought effects on prey populations. Both direct and indirect effects can place considerable stress on organisms by increasing mortality and decreasing reproduction and overall body condition (see Allen et al., 2010; Capehart et al., 2016; Urban, 2015; Zani & Stein, 2018). Limited resources may lead to physiological trade‐offs between various components of individual fitness (Stearns, 1989). These trade‐offs may involve various physiological processes, including growth, reproduction, energy storage, and immune function (Adamo & Lovett, 2011; Bonier et al., 2007; Dupoué et al., 2015; Soler et al., 2003; Werner & Anholt, 1993). Understanding how organisms adapt to ecological pressures such as drought is critical for understanding the mechanisms underlying future impacts of climate change at the level of individual species (Martin et al., 2010; Rohr et al., 2013).

The field of eco‐immunology provides a useful framework with which to study physiological reactions to environmental change in free‐living populations (Downs & Stewart, 2014; Martin et al., 2010; Rohr et al., 2013). Immune function is energetically costly to develop and maintain and can shift in response to resource scarcity, water availability, pathogen exposure, and pollution (see Bowden, 2008; Brusch et al., 2019; Georgiev et al., 2016; Lifjeld et al., 2002; Lochmiller & Deerenberg, 2000; McDade et al., 2016; Vermeulen et al., 2015). In particular, innate or nonspecific immunity—the branch of the immune system which is composed of generalist, preexisting mechanisms of defense—may be a particularly useful indicator of population response to chronic environmental stressors since it is (a) not dependent on specific antigens or current infection and (b) can be compared across periods of time or between different environments (Beutler, 2004; Buehler et al., 2008, 2009; Matson et al., 2005; Riera Romo et al., 2016). Furthermore, there is a growing body of literature that demonstrates an important correlation between innate immunity and environmental situations, such as drought, resource availability, and pollution (Bowden, 2008; Brusch et al., 2019; Georgiev et al., 2016; McDade et al., 2016; Vermeulen et al., 2015). For instance, a study on great‐tit nestlings found a correlation between higher resource abundance and higher investment in immune function (Buehler et al., 2009). Innate immunity has also been negatively correlated to aridity in wild populations of birds (Horrocks et al., 2015). This relationship is not clearly understood, however, since both resource scarcity and pathogen exposure may mediate the correlation between aridity and immunity (Brusch et al., 2019; Horrocks et al., 2015). Such studies indicate that while numerous environmental factors may affect immune system development, the relationship between environmental change and immune function is unclear and much remains to be learned, especially in wild populations (Brusch et al., 2019; Festa‐Bianchet, 1989; Sparkman & Palacios, 2009).

In this seven‐year study, we examined the impact of the recent California drought on innate immunity in two sympatric species of semi‐aquatic garter snakes, *Thamnophis sirtalis* and *T. elegans* from four populations around Eagle Lake in northern California. Short‐term variation in precipitation impacts anuran breeding success and thus directly affects the abundance of amphibians in wetland habitats in this system (Miller et al., 2014); indeed, there is a trend across North America for amphibian communities to be especially sensitive to precipitation immediately prior to breeding, and for warmer winters to have generally negative impacts in western montane habitats (Miller et al. 2018). Previous research has shown that variation in precipitation also has a negative effect on pregnancy and survival rates in garter snakes, most likely via variation in prey availability (Miller et al. 2011; Miller et al., 2014). There is some evidence that differences in prey availability among habitats may explain variation in innate and acquired immunity for snakes at Eagle Lake (Palacios et al., ,,^2011^, ^2013^; Sparkman & Palacios, 2009), but no study has yet tested for temporal patterns in immunity that track differences in climate. Furthermore, it is unclear whether physiological investment in immune function is shaped by climate of the current year or the previous year. It is well‐known that reproduction in some species, particularly snakes, may experience time‐lagged effects (Bonnet et al., 1998; Gregory & Skebo, 1998; Lourdais et al., 2002), and recent research suggests a time‐lag effect of pika abundance in response to drought (Johnston et al., 2019). Thus, it is of interest to determine whether immune function can also experience a time‐lagged response to drought.

To examine the effect of drought conditions on innate immunity, we measured three indices of innate immunity from blood and plasma samples collected in the field: bactericidal competence (BC) of blood, levels of natural antibodies (NABs), and complement‐mediated lysis (CL). These indices, developed for domestic birds by Matson et al. (2005) and Matson et al. (2006) and adapted for garter snakes by Sparkman and Palacios (2009), quantify the strength of immune defensive response in several key components of innate immunity. More specifically, the BC assay examines the ability of blood and plasma samples to mobilize against novel pathogen exposure (in this case *Escherichia coli)*, and the NABs and CL assays assess hemagglutination and hemolysis strength of blood plasma after novel exposure to novel foreign red blood cells (Matson et al., 2005, 2006). Combined together, the results of the NABs, CL, and BC assays quantify immune strength for different aspects of the innate immune response and provide an overall indicator of several key aspects of innate immunity in individuals from our study populations across the years of the California drought. We tested for evidence that these three major indices of innate immunity were affected by variation in annual climate (temperature, snow pack precipitation, and drought indices) from 2012–2018. We predicted that immune function would be lower during the years characterized by drought conditions (2012–2015), when prey and moisture availability were low. Furthermore, we tested for evidence of a time‐lagged effect of climate on innate immunity, consistent with a capital‐breeding reproductive strategy.

## 2 MATERIALS AND METHODS

### 2.1 Study system

Two species of garter snakes (*Thamnophis elegans* and *T. sirtalis*) were studied from four populations (M1, M3, M4, and L1—see Matson et al., 2006 and Gangloff et al., 2020 for population details) at a well‐known study system around Eagle Lake in Northern California. *T. elegans* and *T. sirtalis* are both found in the seasonally flooded, high‐elevation meadows (M1, M3, and M4) around Eagle Lake and rely primarily on a diet of anurans, leeches, and occasionally small mammals—with a particular reliance on the Sierran tree frog (*Pseudacris sierra*) (Kephart, 1982). However, only *T. elegans* are commonly found along the rocky lakeshore of Eagle Lake (L1 in this study), and these populations are primarily dependent on shoals of small minnows such as tui chub (*Gila bicolor)* and speckled dace (*Rhinichthys osculus*) as prey (Kephart, 1982).

During the severest years of the California drought (2012–2015), several populations of garter snakes in our study experienced severe decline. Data from our long‐term studies indicate that 7 of the 15 total study populations in the area were extirpated, predominantly those around the lakeshore (A. M. Bronikowski, *unpublished data*). During the years of the drought, decreased snowfall and spring precipitation caused lake levels at Eagle Lake to decrease rapidly (D. Willis, personal communication, 6 May 2019), exposing large muddy banks which increased the distance between sheltering/basking and foraging areas and thus increasing snake exposure to predators. In addition, prey populations have also fluctuated substantially for meadow populations; for instance, in 2014 amphibian breeding was impeded in several meadows due to severely dry conditions in the normally flooded meadows.

### 2.2 Data collection

Snakes were surveyed during the month of June during the summers of 2012 to 2018. All snakes were caught by hand and bled intravenously through the tail. Less than 1% bodyweight of blood (50 to 300 μl) was collected with heparinized syringes and kept on ice until centrifuged to separate the cellular components of blood from plasma, which was stored in liquid nitrogen for later use in immunocompetence assays. Sample sizes for each assay are as follows: *T.elegans*: BC *n* = 405, NABs *n* = 359, CL *n* = 294; *T. sirtalis:* BC *n* = 170, NABs *n* = 188, CL *n* = 163. For overall sample sizes by year and population see Table 1.

**TABLE 1:** Number of individuals sampled per species per population per year, and total across all populations per species per year

	Year	L1	M1	M3	M4	TOTAL
*T. elegans*	2012	0	23	8	2	10
	2013	20	22	14	4	60
	2014	10	16	3	6	35
	2015	27	23	19	6	75
	2016	20	30	25	10	85
	2017	20	24	20	15	79
	2018	20	21	19	8	68
*T. sirtalis*	2012	.	3	12	6	18
	2013	.	4	11	13	28
	2014	.	0	20	0	20
	2015	.	6	6	3	15
	2016	.	10	14	6	30
	2017	.	12	18	1	31
	2018	.	19	20	16	55

Cells with '.' indicate that no snakes were captured from that population in that year.

Body weight, sex, species, and date of capture were recorded for each snake. Snout‐to‐vent length (SVL) was also collected and can be considered a proxy for snake age since body length is correlated with age in this system (Bronikowski & Arnold, 1999). We also recorded presence/absence of tail trematode infection by looking for swelling, pinkness, or other abnormalities on the tail (Uhrig et al., 2015).

### 2.3 Climate data

**FIGURE 1:**
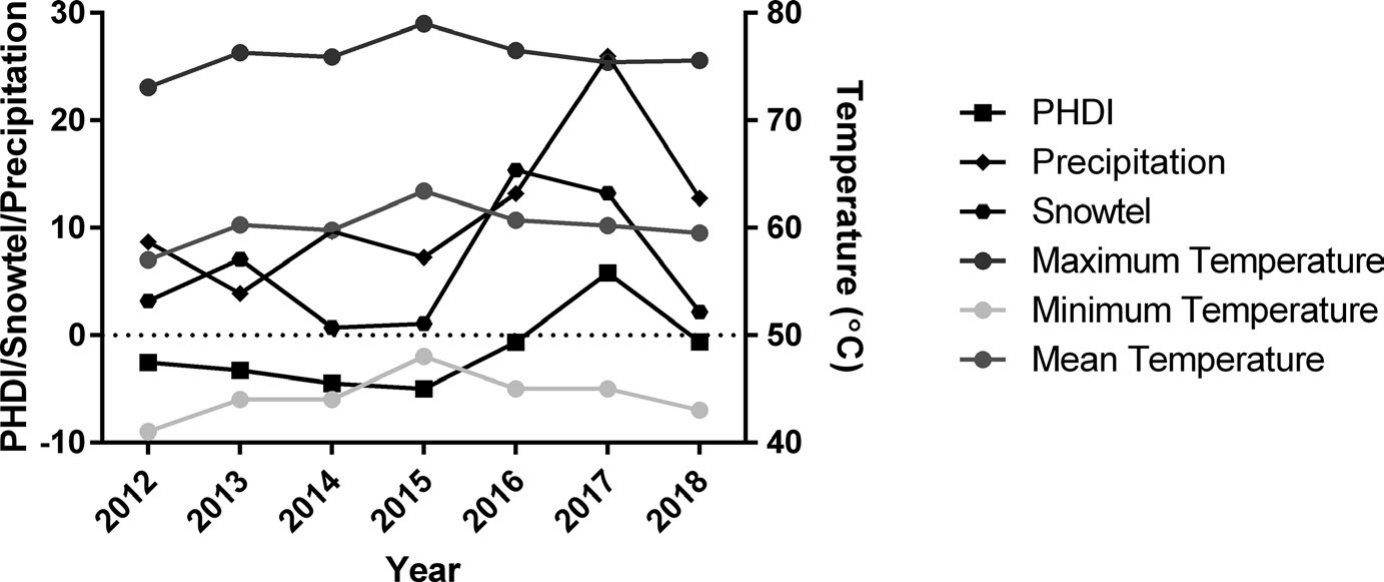
Annual variation in climate variables across the seven years of study. SNOWTEL and precipitation units are as reported in inches. PHDI is an index without units

Climate data for California Climate Division 3 were obtained from the National Oceanic and Atmospheric Administration (NOAA) National Climate Data Center (NCDC) (Vose et al., 2014). For this study, we examined levels of snow telemetry (SNOTEL), a measure of snow pack, snow water content, and snow precipitation, and Palmer Hydrological Drought Index (PHDI), a long‐term measure of drought and aridity that also accounts for soil moisture, ground water, and temperature (Jacobi et al., 2013; Palmer, 1965). SNOTEL data were obtained from the nearest station, located on Mt. Adin, and the average of March and April values for each year were used in the analysis. April estimates for PHDI were used, and spring precipitation was defined as the cumulative precipitation between January and May which, along with snow smelt, is the primary source of spring water availability in the montane meadow habitats. We also obtained minimum, maximum, and mean monthly June temperatures, which correspond to the time of peak snake activity and field data collection. We also tested for the possibility of a one‐year time‐lag for the effect of temperature and precipitation on immunity. This was done by creating a field that assigned the climate values of the previous year (year – 1) for each individual entry. Annual climate variables are shown in Figure 1.

### 2.4 Immunological assays

A natural antibody (NABs) and complement‐mediated lysis (CL) assay, developed by Matson et al., (2005), and modified for garter snakes by Sparkman and Palacios (2009) was performed to assess hemagglutination and hemolysis strength of blood plasma. Serial twofold dilutions of 10 µl plasma were made with Dulbecco's phosphate‐buffered saline 14,190,144 (Thermo Fisher Scientific, Santa Barbara, USA). Sheep red blood cells (SRBC, cat # SBA‐050) in Alsevers was centrifuged four times for 10 min at 500 *g* and rinsed with PBS, and 10 µl 2% SRBC dilution was pipetted into each well (Hemostat Laboratories, Dixon, USA). Plates were incubated at 28°C for 60 min and then scored immediately for lysis and agglutination. On each plate, 1,000 µl PBS and 10 µl SRBC served as a negative control. Scores were given for the highest well that displayed hemagglutination or lysis respectively, and intermediate wells were given half scores.

Bactericidal competence was assessed using a method developed by Matson et al. (2006) and adapted for garter snakes by Sparkman and Palacios (2009). One pellet of dehydrated *Escherichia coli* ATCC 8,739 was dissolved in 40 ml PBS and incubated at 34°C for 30 min (American Type Culture Collection, Manassas, USA). For each *T. elegans* plasma sample*,* a 1:80 plasma to PBS dilution was made, whereas for *T. sirtalis,* a 1:83 dilution was prepared. A 1:25 dilution the *E. coli* dilution was made with PBS and 10 µl of this solution was added to each plasma dilution. Samples were assayed in duplicate using 50 µl aliquots on each plate and sterilizing a spreading slide with 70% EtOH in between application. Plates were left to dry for 20 min and then incubated overnight at 37°C, after which colonies were counted manually. The intra‐assay coefficient of variation for each assay/species is as follows: BC—*T. elegans* 13.9, *T. sirtalis* 14.6; NABs—*T. elegans* 10.8, *T. sirtalis* 7.5; CL—*T. elegans* 11.8, *T. sirtalis* 11.5.

### 2.5 Statistical analyses

We tested for correlations between our indices of innate immunity and climate using simple correlation analysis. We conducted a mixed model analysis of covariance (ANCOVA) for each immune variable for both *T. sirtalis* and *T. elegans*. All four assumptions for ANCOVA were met (but see discussion of multicollinearity below). The dependent variables—bactericidal competence (BC), natural antibodies (NABs), and complement‐mediated lysis (CL)—were tested for effects of SVL, body condition (defined as the residuals of the regression of mass on SVL), day of year, PHDI, SNOTEL, mean/max/min temperature and spring precipitation as covariates and reproductive status (male/nongravid female/gravid female), source population (L1/M1/M3/M4), and parasites (presence/absence of tail trematodes) as fixed effects. We also tested for interactions between climate variables and source population. Year was included in each model as a random effect, as climate variables were not independent among years, and we made no a priori predictions for our models regarding which years were drought and which were nondrought. All variables were added one by one in a stepwise fashion and those with *p* ≥ .2 were dropped from the model, as this indicated that they explained little or no variation in immune function. Note that some of our temperature variables were strongly correlated (see *Results)*. To confirm that the order in which variables were introduced did not affect our outcome and to address any potential concerns with multicollinearity, we performed this process by adding and removing variables in different orders such that every possible combination of variables was ultimately considered. Regardless of the model selection method, we arrived at the same top models. Note that none of the top models contained combinations of explanatory variables that exhibited collinearity. Significance was assessed at an α‐level of *p* = .05. Significant differences among groups were assessed post hoc using Tukey's HSD test. All analyses were conducted with JMP 14.1.0 (SAS Institute, Inc), and figures were constructed using Prism 8.0.2 (GraphPad Software, Inc.).

## 3 RESULTS

There were significant positive correlations between each of our three indices of innate immunity. As previously documented (Sparkman & Palacios, 2009), NABs were significantly positively correlated to CL titers in both species (*T. elegans*: *r* = 0.73, *p* < .0001; *T. sirtalis*: *r* = 0.74, *p* < .0001). Similarly, though the relationship was less pronounced, both NABs and CL were both also significantly positively correlated to BC in both species (*T. elegans* NABs: *r* = 0.41, *p* <.0001; *T. sirtalis* NABs: r = 0.45, *p* <.0001; *T. elegans* CL: *r* = 0.38, *p* < .0001; *T. sirtalis* CL: *r* = 0.34, *p* < .0001). Pairwise correlations among all environmental variables indicated that only the three temperatures variables were significantly correlated to each other (*r* = 0.95–0.99, all *p* < .0001). All other pairwise correlations were nonsignificant (*p* > .1).

**FIGURE 2:**
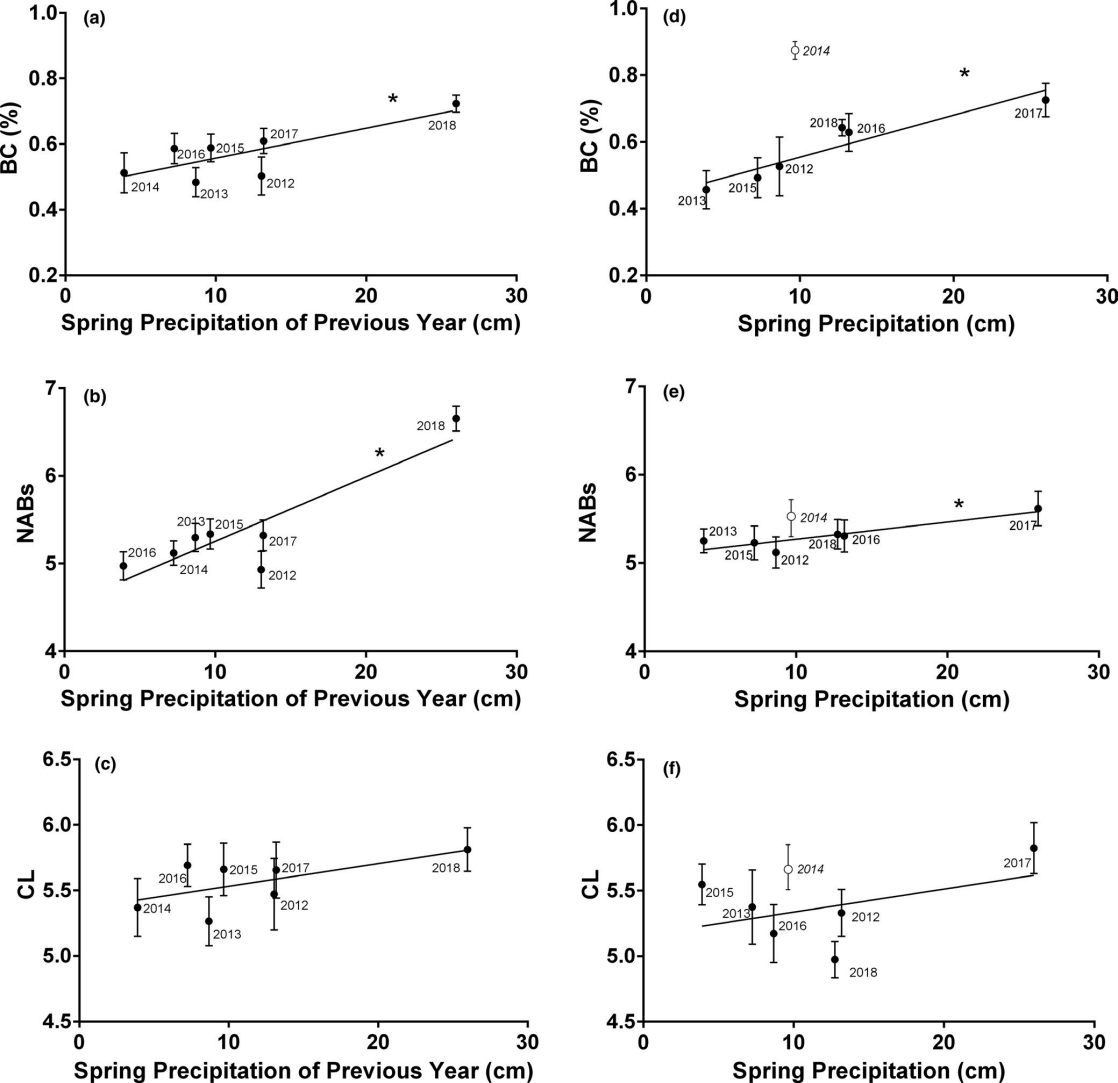
Relationship between indices of innate immunity and spring precipitation from 2012 to 2018. *T. elegans* (a–c) and *T. sirtalis* (d–f). Though the analysis presented in Table 2 is conducted with precipitation as a continuous covariate and year as a random effect, least square means (cm) and standard errors for each year from the final model are depicted here with respect to spring precipitation of the previous year for *T. elegans* and the current year for *T. sirtalis*. Note that *T. sirtalis* data from 2014 were excluded from the final analysis (see Results), but mean 2014 values are indicated with an open marker. Asterisks indicate significant correlations

**FIGURE 3:**
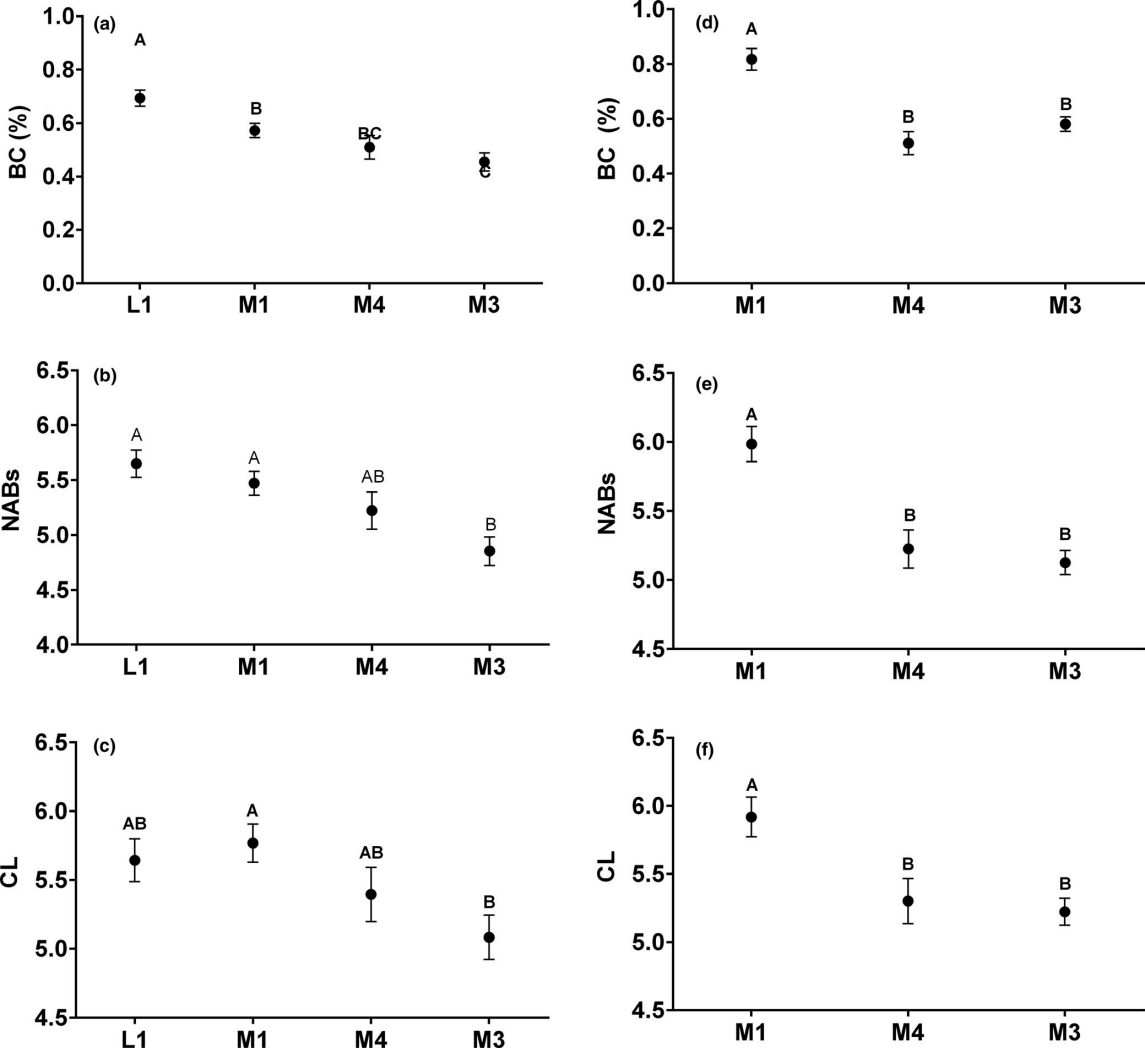
Differences among populations with respect to NABs, CL, and BC for *T. elegans* (a–c) and *T. sirtalis* (d–f). Least square means and standard errors of the means are shown. Different letters indicate significant differences between groups

**FIGURE 4:**
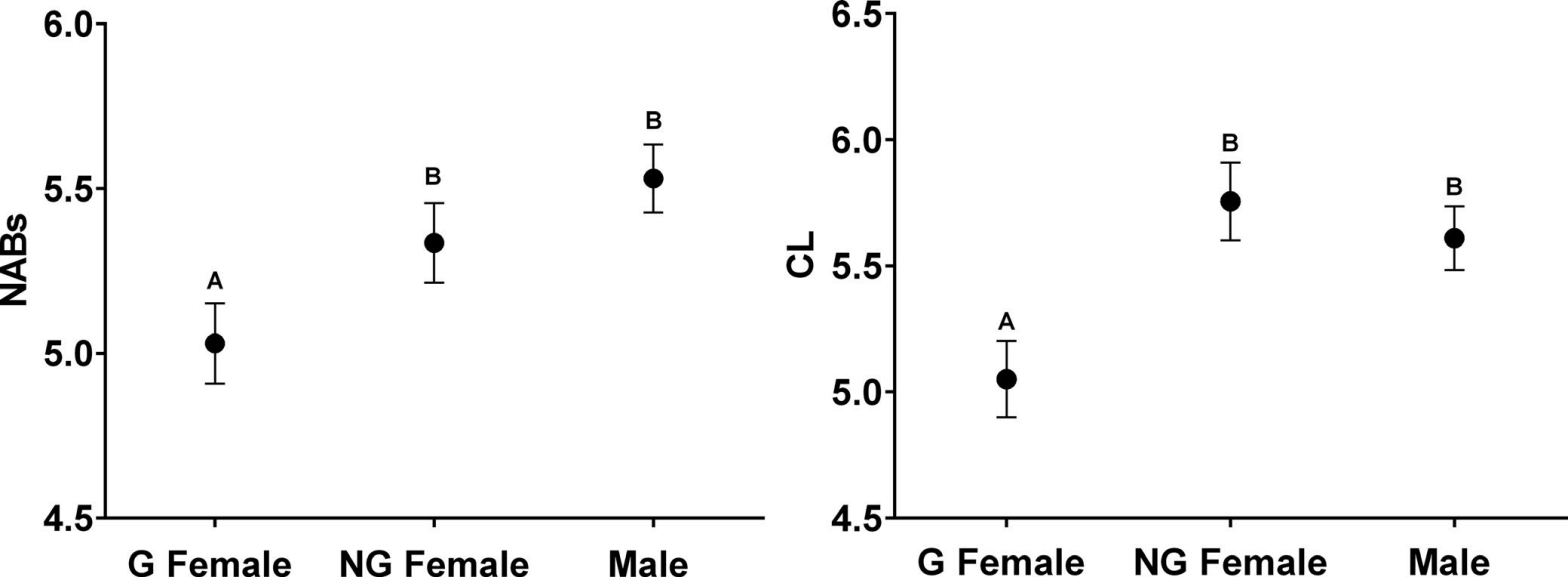
Differences in NABs and CL in *T. elegans* with respect to differences in reproductive status (gravid (G) female, nongravid (NG) female or male). Least square means and standard errors of the means are shown. Different letters indicate significant differences between groups

The final model for each *T. elegans* index of immunity included source population and year as a random effect (Table 2). Both BC and NABs were significantly correlated to spring precipitation of the former year (Figure 2a, b; Table 2). No climate variables were significantly correlated to CL; however, PHDI was retained in the model as it showed a *p* < .1 (Table 2). Differences among populations were largely consistent across immune indices, with L1 and M1 consistently at the high end, M4 intermediate, and M3 consistently at the low end (Figure 3a–c). Additionally, reproductive status (i.e., gravid female, nongravid female, or male) was a significant effect in the final model for NABs and CL, with gravid females having significantly lower NABs and CL scores than both nongravid females and males (Table 2; Figure 4).

**TABLE 2:** Results of mixed model analysis of immune variables with year included as a random effect

Species	Dependent variable	Explanatory variable	*df*	*F*	*p*
*T. elegans*	Bactericidal competence	Source Population	3, 401	10.73	<.0001
		Precipitation ‐ 1	1, 4	12.24	.020
	Natural antibodies	Source Population	3, 342	8.09	<.0001
		Precipitation ‐ 1	1, 2	72.48	.027
		Reproductive Status	2, 222	7.46	.001
	Complement‐mediated lysis	Source Population	3, 283	3.55	.015
		PHDI	1,4	4.58	.093
		Reproductive Status	2, 275	9.13	.0001
*T. sirtalis*	Bactericidal competence	Source Population	1, 110	10.67	<.0001
		Precipitation	1, 2	25.99	.033
	Natural antibodies	Source Population	3, 161	7.72	.0002
		Precipitation	1, 10	6.83	.027
	Complement‐mediated lysis	Source Population	3, 157	5.38	.002

As with *T. elegans*, the final model for each *T. sirtalis* index of immunity included source population and year as a random effect (Table 2). In our first round of analyses, it appeared that the only relationship between annual climate and innate immunity in *T. sirtalis* occurred with respect to precipitation in the current year and NABs. However, we noted that 2014 (the most severe drought year) was an unusual year in that we only had *T. sirtalis* represented from a single population, which appeared to be exhibiting unusually high BC values (see Figure 2d). Thus, we also ran our analyses excluding 2014 and found that in this case BC, like NABs, exhibited a significant, positive correlation with precipitation the current year (Table 2, Figure 2d–e). Consistent with *T. elegans*, M1 consistently had the highest levels for each measure of innate immunity, whereas both M4 and M3 levels were low (Figure 3). There was no trend for reduced immunity in gravid *T. sirtalis* females for any of the three indices.

Other variables considered—SVL, body condition, day of year, SNOTEL, mean/min/max temperature, incidence of parasites, and interactions between climate variables and source population—did not have *p* < .2 in any model for any immune index for either species.

## 4 DISCUSSION

Previous work has shown that environmental characteristics, including climate, pathogen intensity, resource abundance, and pollution can impact indices of innate immunity (Bowden, 2008; Brusch et al., 2019; Georgiev et al., 2016; McDade et al., 2016; Vermeulen et al., 2015). Consequently, we predicted that annual climatic variation associated with drought, which has already been shown to decrease body condition and reproductive success in reptiles, would result in a decrease in innate immunity in garter snakes (Allen et al., 2010; Capehart et al., 2016; Urban, 2015; Zani & Stein, 2018). Consistent with our expectations, we found that, in addition to showing consistent patterns of variation both within and across populations, innate immunity was associated with annual spring precipitation in both species of sympatric garter snake in our study—albeit in a species‐specific manner.

We tested for the influence of seven regional climate variables on innate immunity, including annual indices of snowpack; spring precipitation; mean, maximum, and minimum June temperature; and a drought index, PHDI. Only spring precipitation, however, explained significant variation in immune function in our study. Two of three measures of immunity—BC and NABs—were positively correlated with spring precipitation in *T. elegans* and *T. sirtalis* (though note that BC was only significant for the latter when unusually high values during 2014 were excluded from the analysis). Interestingly, while BC and NABs were significantly associated with spring precipitation in the current year for *T. sirtalis*, *T. elegans* exhibited a time‐lagged pattern, such that immunity was correlated to spring precipitation of the previous year (Table 2; Figure 2). Reproductive investment in garter snakes and other species may depend primarily on the resource capital acquired a year prior (Bonnet et al., 1998; Gregory & Skebo, 1998; Lourdais et al., 2002; Sabrina et al., 2009). The finding that both BC and NAB levels in *T. elegans* were significantly explained by precipitation levels of the previous year indicates that stored capital may also influence immune function in some species (Table 2). To our knowledge, this is the first time a time‐lagged effect of climate has been demonstrated in immunological—or indeed, any physiological—study in the wild. This finding complements other recent work in pika (*Ochotona princeps*) showing the delayed effect of drought on population mortality (Johnston et al., 2019) and calls for investigation into investment in immune function as a possible mechanism for this effect.

We are uncertain whether populations of the two species in our study differ in the extent to which they are capital or income breeders. It is clear that *T. elegans* demography is very sensitive to the conditions of the previous year such as variation in annual spring precipitation, which may limit access to water or decrease prey availability. This in turn may lead to reductions in probability of individual reproduction, rates of growth, individual condition, and survival the following year (Dinsmore, 2019; Miller et al., 2011; Miller et al., 2018). In contrast, there is some evidence that although *T. sirtalis* are also sensitive to conditions in the previous year, they are less so. For example, over‐winter survival is not correlated with conditions in the previous summer and nearly all adult females reproduce every year regardless of conditions (Dinsmore, 2019). We are currently pursuing additional analyses of climate effects on survival, reproduction, and stress physiology that will be of great interest to combine with our findings regarding the presence of a time‐lagged effect of climate on *T. elegans* but not *T. sirtalis*.

Our findings prompt the question of how spring precipitation might be affecting innate immunity, if this correlation reflects causation. Climate change may affect organisms either directly, through effects on survival, growth, reproduction and/or dispersal, or indirectly, via impacts on community dynamics, such as competitive and predator‐prey interactions, pathogen‐host relationships, and habitat characteristics including vegetation and hydrology (reviewed in Blaustein et al., 2010). In our system, we have ample evidence that water availability significantly affects the abundance of anuran prey, such that the driest years may be characterized by little to no prey availability (Miller et al., 2011, Miller et al., 2014). However, beyond evaluations of tail trematode infections, we know little regarding the complex nature of predatory and pathogenic interactions that could be exerting stress in concert with reduced prey in our system (Bowden, 2008; Brusch et al., 2019; Georgiev et al., 2016; McDade et al., 2016; Vermeulen et al., 2015; Zani & Stein, 2018). Regardless of the mechanism, reduced innate immunity could have detrimental consequences for survival and population persistence (Charbonnel et al., 2008; Møller & Saino, 2004). Lower immunity during dry years or years following dry years could be the result of a life‐history trade‐off, where investment in other factors such as reproduction or growth is being prioritized over physiological correlates of survival, such as immune function (Sheldon & Verhulst, 1996). Recent work in frogs (*Rana pipiens*), for instance, has shown evidence of trade‐offs between growth, survival, and immune function in the context of rapid pond drying (Brannelly et al., 2019). Alternatively, if resources are sufficiently limited during dry years, we might expect reduced investment in all measurable life‐history outcomes, such that individuals are not only suffering reduced immune function, but also experiencing reductions in other critical life‐history traits such as growth and reproduction.

While previous work has shown variation in immune function associated with variation in resource availability, pathogen exposure, or other mediating ecological variables (Buehler et al., 2009; Horrocks et al., 2015; Pigeon et al., 2013; Sandland & Minchella, 2003; Vermeulen et al., 2015), obtaining high‐quality data on spatial and temporal heterogeneity in environmental variables is in fact one of the major challenges in the field of eco‐immunology—or “macroimmunology” as it has been recently termed (Becker et al. 2020). We are currently pursuing a fully linked analysis exploring repeated measures of immune function and other physiological and genetic endpoints in relationship to growth, reproduction and survival, which will help illuminate the complex effects of climate and the interplay of variables at each level of biological organization. The question of whether climate influences immune function directly, or via community dynamics, however, will require intensive and strategic field work in the future that quantifies temporal and spatial changes in local climate, hydrology, vegetation, prey availability, additional pathogens, and predator‐mediated stressors. We would anticipate that immune function as a whole is in fact a complex function of many or all of these factors, including climate; but which, if any, prove to more strongly and/or directly impact some indices of immune function than others must be ascertained through additional study.

Our study system is a powerful one in which to continue to explore these questions, due to the long‐term nature of the data we do have on well‐differentiated populations of garter snakes distributed across the landscape–and indeed, source population was a significant effect in the final model for each immune variable for both *T. elegans* and *T. sirtalis* in our study (Table 2). Population differences in immunity may have been due to variations in habitat quality and prey availability between our population sites. Of the three populations from which *T. sirtalis* were sampled, all three immune indices were highest at M1 meadow (Figure 4). Compared with the other two population sites, this meadow is the largest and is populated by both minnows and anurans. It has the lowest elevation, is closest to the lakeshore, and is fed by deep streams, arguably making it the most ideal habitat for the snakes. *T. elegans* were sampled from a lakeshore study site (L1) in addition to the three meadow sites. In general, we found that immune function was highest at the lakeshore site, with M1 meadow a close second (Figure 3). The lakeshore habitat may offer the most consistent food source, since drought levels may not have had as severe of an impact on the abundance of minnows in the lake. In general, these findings are compelling in that they suggest that regardless of the plasticity that each population may show in innate immunity across years, there may also be fixed differences between habitats that have independently resulted in consistent patterns of innate immunity in the two snake species that reside there. Whether this reflects plastic responses to habitat characteristics for both species, or selection resulting in convergent genetic differentiation in immune function remains to be determined.

Neither body size/age nor condition appeared to affect these indices of immune function in this study, which is largely consistent with prior work in this system (Palacios et al. 2011; Palacios et al., 2013; Palacios & Bronikowski, 2017—but see Sparkman & Palacios, 2009 for a relationship between NABS/CL and SVL). However, our results do suggest that gravid *T. elegans* females have lower innate immunity than nongravid females and males, although this pattern was significant only for NAB and CL scores (Table 2, Figure 4), and was not present in *T. sirtalis*. Previous work in this system has shown reduced adaptive immunity for gravid females in this system (Palacios & Bronikowski, 2017). Similar trends have been observed in mammals and birds, since females experience physiological trade‐offs during reproduction, and may invest less energy into immunity due to the high energetic costs of reproduction (Festa‐Bianchet, 1989; Neggazi et al., 2016; but see Neggazi et al., 2016). Our results are also consistent with other work on reptiles that has found that reproductive female lizards experience severe decline in body condition during drought (Zani & Stein, 2018). However, it is worth noting that even in this study of two closely related species living in sympatry, only one showed lower immune function during reproduction, indicating that the ability to predict reduced immune function in gravid individuals in any system without prior empirical evidence to that effect may be limited.

In this study, we provide evidence that drought conditions may lead to a reduction in immune function in a similar but distinctive manner in multiple populations of two closely related species living in the same habitats and experiencing the same fluctuations in annual climate. In addition, we provide evidence of reliance on capital for immune investment in *T. elegans*, reminiscent of a capital‐breeding strategy (Gregory & Skebo, 1998; Lourdais et al., 2002). Together, these findings have important implications for the impact of climate change‐induced drought on the physiological adaptation and survival of reptiles and other species. As droughts become more frequent and severe, threatened species may undergo physiological challenges and incur physiological costs that leave them vulnerable to diseases and parasites, leading to mortality and population decline. Our work brings us closer to understanding the complex dynamics of this system in free‐ranging species, with compelling ramifications for future research on the mechanisms underlying species‐specific responses to climate change worldwide.

## CONFLICT OF INTEREST

The authors declare no conflict of interest.

## AUTHOR CONTRIBUTIONS

**Lucia L. Combrink:** Conceptualization (supporting); Data curation (lead); Formal analysis (equal); Visualization (equal); Writing‐original draft (lead); Writing‐review & editing (lead). **Anne M. Bronikowski:** Funding acquisition (equal); Methodology (supporting); Writing‐review & editing (supporting). **David A. W. Miller:** Funding acquisition (equal); Writing‐review & editing (supporting). **Amanda M. Sparkman:** Conceptualization (lead); Data curation (supporting); Formal analysis (lead); Funding acquisition (equal); Investigation (lead); Methodology (lead); Project administration (lead); Software (lead); Supervision (lead); Visualization (equal); Writing‐original draft (supporting); Writing‐review & editing (supporting).

## Data Availability

Data can be accessed from the Dryad Digital Repository https://doi.org/10.5061/dryad.xksn02vcj (Combrink et al., 2020).

## References

[ece37273-bib-0001] Adamo, S. A. , & Lovett, M. M. (2011). Some like it hot: The effects of climate change on reproduction, immune function and disease resistance in the cricket *Gryllus texensis* . Journal of Experimental Biology, 214(12), 1997–2004. 10.1242/jeb.056531 21613515

[ece37273-bib-0002] Allen, C. D. , Macalady, A. K. , Chenchouni, H. , Bachelet, D. , McDowell, N. , Vennetier, M. , Kitzberger, T. , Rigling, A. , Breshears, D. D. , Hogg, E. H. T. , Gonzalez, P. , Fensham, R. , Zhang, Z. , Castro, J. , Demidova, N. , Lim, J.‐H. , Allard, G. , Running, S. W. , Semerci, A. , & Cobb, N. (2010). A global overview of drought and heat‐induced tree mortality reveals emerging climate change risks for forests. Forest Ecology and Management, 259(4), 660–684. 10.1016/j.foreco.2009.09.001

[ece37273-bib-0003] Araújo, M. B. , Thuiller, W. , & Pearson, R. G. (2006). Climate warming and the decline of amphibians and reptiles in Europe. Journal of Biogeography, 33(10), 1712–1728. 10.1111/j.1365-2699.2006.01482.x

[ece37273-bib-0004] Asner, G. P. , Brodrick, P. G. , Anderson, C. B. , Vaughn, N. , Knapp, D. E. , & Martin, R. E. (2016). Progressive forest canopy water loss during the 2012–2015 California drought. Proceedings of the National Academy of Sciences, 113(2), E249–E255. 10.1073/pnas.1523397113 PMC4720336 26712020

[ece37273-bib-0005] Bales, R. C. , Goulden, M. L. , Hunsaker, C. T. , Conklin, M. H. , Hartsough, P. C. , O’Geen, A. T. , Hopmans, J. W. , & Safeeq, M. (2018). Mechanisms controlling the impact of multi‐year drought on mountain hydrology. Scientific Reports, 8(1), 690. 10.1038/s41598-017-19007-0 29330378 PMC5766567

[ece37273-bib-0006] Barnosky, A. D. , Matzke, N. , Tomiya, S. , Wogan, G. O. U. , Swartz, B. , Quental, T. B. , Marshall, C. , McGuire, J. L. , Lindsey, E. L. , Maguire, K. C. , Mersey, B. , & Ferrer, E. A. (2011). Has the Earth’s sixth mass extinction already arrived? Nature, 471(7336), 51–57. 10.1038/nature09678 21368823

[ece37273-bib-0007] Becker, D. J. , Albery, G. F. , Kessler, M. K. , Lunn, T. J. , Falvo, C. A. , Czirják, G. Á. , Martin, L. B. , & Plowright, R. K. (2020). Macroimmunology: The drivers and consequences of spatial patterns in wildlife immune defence. Journal of Animal Ecology, 89(4), 972–995. 10.1111/1365-2656.13166 PMC7138727 31856309

[ece37273-bib-0008] Benjaminsen, T. A. , & Hiernaux, P. (2019). From desiccation to global climate change: A history of the desertification narrative in the west African sahel, 1900–2018. Global Environment, 12(1), 206–236. 10.3197/ge.2019.120109

[ece37273-bib-0009] Beutler, B. (2004). Innate immunity: An overview. Molecular Immunology, 40(12), 845–859. 10.1016/j.molimm.2003.10.005 14698223

[ece37273-bib-0010] Blaustein, A. R. , Walls, S. C. , Bancroft, B. A. , Lawler, J. J. , Searle, C. L. , & Gervasi, S. S. (2010). Direct and indirect effects of climate change on amphibian populations. Diversity, 2(2), 281–313. 10.3390/d2020281

[ece37273-bib-0011] Bonier, F. , Martin, P. R. , Jensen, J. P. , Butler, L. K. , Ramenofsky, M. , & Wingfield, J. C. (2007). Pre‐migratory life history stages of juvenile arctic birds: Costs, constraints, and trade‐offs. Ecology, 88(11), 2729–2735. 10.1890/07-0696.1 18051640

[ece37273-bib-0012] Bonnet, X. , Bradshaw, D. , & Shine, R. (1998). Capital versus income breeding: An ectothermic perspective. Oikos, 83(2), 333–342. 10.2307/3546846

[ece37273-bib-0013] Bowden, T. J. (2008). Modulation of the immune system of fish by their environment. Fish & Shellfish Immunology, 25(4), 373–383. 10.1016/j.fsi.2008.03.017 18562213

[ece37273-bib-0014] Brannelly, L. A. , Ohmer, M. E. , Saenz, V. , & Richards‐Zawacki, C. L. (2019). Effects of hydroperiod on growth, development, survival and immune defences in a temperate amphibian. Functional Ecology, 33(10), 1952–1961. 10.1111/1365-2435.13419

[ece37273-bib-0015] Bronikowski, A. M. , & Arnold, S. J. (1999). The evolutionary ecology of life history variation in the garter snake *Thamnophis elegans* . Ecology, 80(7), 2314–2325. 10.1890/08-0850.1 19341142

[ece37273-bib-0016] Brusch, G. A. , Heulin, B. , & DeNardo, D. F. (2019). Dehydration during egg production alters egg composition and yolk immune function. Comparative Biochemistry and Physiology Part A: Molecular & Integrative Physiology, 227, 68–74. 10.1016/j.cbpa.2018.10.006 30300746

[ece37273-bib-0017] Buehler, D. M. , Piersma, T. , Matson, K. , & Tieleman, B. I. (2008). Seasonal redistribution of immune function in a migrant shorebird: Annual‐cycle effects override adjustments to thermal regime. The American Naturalist, 172(6), 783–796. 10.1086/592865 18999941

[ece37273-bib-0018] Buehler, D. M. , Tieleman, B. I. , & Piersma, T. (2009). Age and environment affect constitutive immune function in red knots (*Calidris canutus*). Journal of Ornithology, 150(4), 815–825. 10.1007/s10336-009-0402-6

[ece37273-bib-0019] Capehart, G. D. , Escallón, C. , Vernasco, B. J. , Moore, I. T. , & Taylor, E. N. (2016). No drought about it: Effects of supplemental hydration on the ecology, behavior, and physiology of free‐ranging rattlesnakes. Journal of Arid Environments, 134, 79–86. 10.1016/j.jaridenv.2016.06.018

[ece37273-bib-0020] Carnicer, J. , Coll, M. , Ninyerola, M. , Pons, X. , Sanchez, G. , & Penuelas, J. (2011). Widespread crown condition decline, food web disruption, and amplified tree mortality with increased climate change‐type drought. Proceedings of the National Academy of Sciences, 108(4), 1474–1478. 10.1073/pnas.1010070108 PMC3029725 21220333

[ece37273-bib-0021] Charbonnel, N. , Chaval, Y. , Berthier, K. , Deter, J. , Morand, S. , Palme, R. , & Cosson, J. F. (2008). Stress and demographic decline: A potential effect mediated by impairment of reproduction and immune function in cyclic vole populations. Physiological and Biochemical Zoology, 81(1), 63–73. 10.1086/523306 18040973

[ece37273-bib-0022] Chen, I. C. , Hill, J. K. , Ohlemüller, R. , Roy, D. B. , & Thomas, C. D. (2011). Rapid range shifts of species associated with high levels of climate warming. Science, 333(6045), 1024–1026. 10.1126/science.1206432 21852500

[ece37273-bib-0023] Combrink, L. L. , Bronikowski, A. M. , Miller, D. A. , & Sparkman, A. M. (2020). Data from: Current and Time‐Lagged Effects of Climate on Innate Immunity in Two Sympatric Snake Species. Dryad Digital Repository. 10.5061/dryad.xksn02vcj PMC8019058 33841780

[ece37273-bib-0024] Corn, P. S. (2005). Climate change and amphibians. Animal Biodiversity and Conservation, 28(1), 59–67.

[ece37273-bib-0025] Dinsmore, C. R. (2019). The influence of food availability on life history trajectories. Masters Thesis. Pennsylvania State University.

[ece37273-bib-0026] Downs, C. J. , & Stewart, K. M. (2014). A primer in ecoimmunology and immunology for wildlife research and management. California Fish and Game, 100(3), 371–395.

[ece37273-bib-0027] Drost, C. A. , & Fellers, G. M. (1996). Collapse of a regional frog fauna in the Yosemite area of the California Sierra Nevada, USA. Conservation Biology, 10(2), 414–425. 10.1046/j.1523-1739.1996.10020414.x

[ece37273-bib-0028] Dupoué, A. , Brischoux, F. , Angelier, F. , DeNardo, D. F. , Wright, C. D. , & Lourdais, O. (2015). Intergenerational trade‐off for water may induce a mother–offspring conflict in favour of embryos in a viviparous snake. Functional Ecology, 29(3), 414–422. 10.1111/1365-2435.12349

[ece37273-bib-0029] Fellers, G. M. , & Drost, C. A. (1993). Disappearance of the Cascades frog Rana cascadae at the southern end of its range, California, USA. Biological Conservation, 65(2), 177–181. 10.1016/0006-3207(93)90447-9

[ece37273-bib-0030] Festa‐Bianchet, M. (1989). Individual differences, parasites, and the costs of reproduction for bighorn ewes (*Ovis canadensis*). The Journal of Animal Ecology, 785–795, 10.2307/5124

[ece37273-bib-0031] Gangloff, E. J. , Schwartz, T. S. , Klabacka, R. , Huebschman, N. , Liu, A. Y. , & Bronikowski, A. M. (2020). Mitochondria as central characters in a complex narrative: Linking genomics, energetics, and pace‐of‐life in natural populations of garter snakes. Experimental Gerontology, 110967. 10.1016/j.exger.2020.110967 32387125

[ece37273-bib-0032] Georgiev, A. V. , Kuzawa, C. W. , & McDade, T. W. (2016). Early developmental exposures shape trade‐offs between acquired and innate immunity in humans. Evolution, Medicine, and Public Health, 2016(1), 256–269. 10.1093/emph/eow022 PMC4996124 27530543

[ece37273-bib-0033] Gregory, P. T. , & Skebo, K. M. (1998). Trade‐offs between reproductive traits and the influence of food intake during pregnancy in the garter snake, *Thamnophis Elegans* . The American Naturalist, 151(5), 477–486. 10.1086/286134 18811321

[ece37273-bib-0034] Griffin, D. , & Anchukaitis, K. J. (2014). How unusual is the 2012–2014 California drought? Geophysical Research Letters, 41(24), 9017–9023. 10.1002/2014GL062433

[ece37273-bib-0035] Horrocks, N. P. C. , Hegemann, A. , Ostrowski, S. , Ndithia, H. , Shobrak, M. , Williams, J. B. , Matson, K. D. , & Tieleman, B. I. (2015). Environmental proxies of antigen exposure explain variation in immune investment better than indices of pace of life. Oecologia, 177(1), 281–290. 10.1007/s00442-014-3136-y 25385541

[ece37273-bib-0036] Huang, J. , Yu, H. , Guan, X. , Wang, G. , & Guo, R. (2016). Accelerated dryland expansion under climate change. Nature Climate Change, 6(2), 166. 10.1038/nclimate2837

[ece37273-bib-0037] Huey, R. B. , Deutsch, C. A. , Tewksbury, J. J. , Vitt, L. J. , Hertz, P. E. , Álvarez Pérez, H. J. , & Garland, T. Jr (2009). Why tropical forest lizards are vulnerable to climate warming. Proceedings of the Royal Society B: Biological Sciences, 276(1664), 1939–1948. 10.1098/rspb.2008.1957 PMC2677251 19324762

[ece37273-bib-0038] Jacobi, J. , Perrone, D. , Duncan, L. L. , & Hornberger, G. (2013). A tool for calculating the Palmer drought indices. Water Resources Research, 49(9), 6086–6089. 10.1002/wrcr.20342

[ece37273-bib-0039] Janzen, F. J. , Hoekstra, L. A. , Brooks, R. J. , Carroll, D. M. , Gibbons, J. W. , Greene, J. L. , Iverson, J. B. , Litzgus, J. D. , Michael, E. D. , Parren, S. G. , Roosenburg, W. M. , Strain, G. F. , Tucker, J. K. , & Ultsch, G. R. (2018). Altered spring phenology of North American freshwater turtles and the importance of representative populations. Ecology and Evolution, 8(11), 5815–5827. 10.1002/ece3.4120 29938095 PMC6010881

[ece37273-bib-0040] Johnston, A. N. , Bruggeman, J. E. , Beers, A. T. , Beever, E. A. , Christophersen, R. G. , & Ransom, J. I. (2019). Ecological consequences of anomalies in atmospheric moisture and snowpack. Ecology, 100(4), e02638. 30710338 10.1002/ecy.2638

[ece37273-bib-0041] Kelley, C. P. , Mohtadi, S. , Cane, M. A. , Seager, R. , & Kushnir, Y. (2015). Climate change in the Fertile Crescent and implications of the recent Syrian drought. Proceedings of the National Academy of Sciences, 112(11), 3241–3246. 10.1073/pnas.1421533112 PMC4371967 25733898

[ece37273-bib-0042] Kephart, D. G. (1982). Microgeographic variation in the diets of garter snakes. Oecologia, 52(2), 287–291. 10.1007/BF00363852 28310523

[ece37273-bib-0043] Lifjeld, J. T. , Dunn, P. O. , & Whittingham, L. A. (2002). Short‐term fluctuations in cellular immunity of tree swallows feeding nestlings. Oecologia, 130(2), 185–190. 10.1007/s004420100798 28547140

[ece37273-bib-0044] Lochmiller, R. L. , & Deerenberg, C. (2000). Trade‐offs in evolutionary immunology: Just what is the cost of immunity? Oikos, 88(1), 87–98. 10.1034/j.1600-0706.2000.880110.x

[ece37273-bib-0045] Lourdais, O. , Bonnet, X. , Shine, R. , DeNardo, D. , Naulleau, G. , & Guillon, M. (2002). Capital‐breeding and reproductive effort in a variable environment: A longitudinal study of a viviparous snake. Journal of Animal Ecology, 71(3), 470–479. 10.1046/j.1365-2656.2002.00612.x

[ece37273-bib-0046] Mann, M. E. , & Gleick, P. H. (2015). Climate change and California drought in the 21st century. Proceedings of the National Academy of Sciences, 112(13), 3858–3859. 10.1073/pnas.1503667112 PMC4386383 25829537

[ece37273-bib-0047] Martin, L. B. , Hopkins, W. A. , Mydlarz, L. D. , & Rohr, J. R. (2010). The effects of anthropogenic global changes on immune functions and disease resistance. Annals of the New York Academy of Sciences, 1195(1), 129. 10.1111/j.1749-6632.2010.05454.x 20536821

[ece37273-bib-0048] Matson, K. D. , Ricklefs, R. E. , & Klasing, K. C. (2005). A hemolysis–hemagglutination assay for characterizing constitutive innate humoral immunity in wild and domestic birds. Developmental & Comparative Immunology, 29(3), 275–286. 10.1016/j.dci.2004.07.006 15572075

[ece37273-bib-0049] Matson, K. D. , Tieleman, B. I. , & Klasing, K. C. (2006). Capture stress and the bactericidal competence of blood and plasma in five species of tropical birds. Physiological and Biochemical Zoology, 79(3), 556–564. 10.1016/j.dci.2004.07.006 16691521

[ece37273-bib-0050] McDade, T. W. , Georgiev, A. V. , & Kuzawa, C. W. (2016). Trade‐offs between acquired and innate immune defenses in humans. Evolution, Medicine, and Public Health, 2016(1), 1–16. 10.1093/emph/eov033 PMC4703052 26739325

[ece37273-bib-0051] Miller, D. A. W. , Grant, E. H. C. , Muths, E. , Amburgey, S. M. , Adams, M. J. , Joseph, M. B. , Waddle, J. H. , Johnson, P. T. J. , Ryan, M. E. , Schmidt, B. R. , Calhoun, D. L. , Davis, C. L. , Fisher, R. N. , Green, D. M. , Hossack, B. R. , Rittenhouse, T. A. G. , Walls, S. C. , Bailey, L. L. , Cruickshank, S. S. , … Sigafus, B. H. (2018). Quantifying climate sensitivity and climate‐driven change in North American amphibian communities. Nature Communications, 9(1), 1–15. 10.1038/s41467-018-06157-6 PMC6156563 30254220

[ece37273-bib-0052] Miller, D. A. W. , Janzen, F. J. , Fellers, G. M. , Kleeman, P. M. , & Bronikowski, A. M. (2014). Biodemography of ectothermic tetrapods provides insights into the evolution and plasticity of mortality patterns. In M. Weinstein , & M. Lane (Eds.), Sociality, Hierarchy, Health: Comparative Biodemography. National Academies Press. 25254285

[ece37273-bib-0053] Miller, D. A. , William, R. C. , Stevan, J. A. , & Anne, M. B. (2011). Stochastic population dynamics in populations of western terrestrial garter snakes with divergent life histories. Ecology, 92(8), 1658–1671. 21905432 10.1890/10-1438.1

[ece37273-bib-0054] Møller, A. P. , & Saino, N. (2004). Immune response and survival. Oikos, 104(2), 299–304. 10.1111/j.0030-1299.2004.12844.x

[ece37273-bib-0055] Neggazi, S. A. , Noreikiene, K. , Öst, M. , & Jaatinen, K. (2016). Reproductive investment is connected to innate immunity in a long‐lived animal. Oecologia, 182(2), 347–356. 10.1007/s00442-016-3657-7 27215635

[ece37273-bib-0056] Palacios, M. G. , & Bronikowski, A. M. (2017). Immune variation during pregnancy suggests immune component‐specific costs of reproduction in a viviparous snake with disparate life‐history strategies. Journal of Experimental Zoology Part A: Ecological and Integrative Physiology, 327(8), 513–522. 10.1002/jez.2137 29356424

[ece37273-bib-0057] Palacios, M. G. , Cunnick, J. E. , & Bronikowski, A. M. (2013). Complex interplay of body condition, life history, and prevailing environment shapes immune defenses of garter snakes in the wild. Physiological and Biochemical Zoology, 86(5), 547–558. 10.1086/672371 23995485

[ece37273-bib-0058] Palacios, M. G. , Sparkman, A. M. , & Bronikowski, A. M. (2011). Developmental plasticity of immune defence in two life‐history ecotypes of the garter snake, *Thamnophis elegans*–a common‐environment experiment. Journal of Animal Ecology, 80(2), 431–437. 10.1111/j.1365-2656.2010.01785.x 21182520

[ece37273-bib-0059] Palmer, W. C. (1965). Meteorological drought, Research Paper No. 45. US Weather Bureau, 58 pp.

[ece37273-bib-0060] Pap, P. L. , Czirják, G. Á. , Vágási, C. I. , Barta, Z. , & Hasselquist, D. (2010). Sexual dimorphism in immune function changes during the annual cycle in house sparrows. Naturwissenschaften, 97(10), 891–901. 10.1007/s00114-010-0706-7 20706704

[ece37273-bib-0061] Pigeon, G. , Bélisle, M. , Garant, D. , Cohen, A. A. , & Pelletier, F. (2013). Ecological immunology in a fluctuating environment: An integrative analysis of tree swallow nestling immune defense. Ecology and Evolution, 3(4), 1091–1103. 10.1002/ece3.504 23610646 PMC3631416

[ece37273-bib-0062] Prugh, L. R. , Deguines, N. , Grinath, J. B. , Suding, K. N. , Bean, W. T. , Stafford, R. , & Brashares, J. S. (2018). Ecological winners and losers of extreme drought in California. Nature Climate Change, 8(9), 819–824. 10.1038/s41558-018-0255-1

[ece37273-bib-0063] Riera Romo, M. , Pérez‐Martínez, D. , & Castillo Ferrer, C. (2016). Innate immunity in vertebrates: An overview. Immunology, 148(2), 125–139. 10.1111/imm.12597 26878338 PMC4863567

[ece37273-bib-0064] Rohr, J. R. , Raffel, T. R. , Blaustein, A. R. , Johnson, P. T. , Paull, S. H. , & Young, S. (2013). Using physiology to understand climate‐driven changes in disease and their implications for conservation. Conservation. Physiology, 1(1), 10.1093/conphys/cot022 PMC4732440 27293606

[ece37273-bib-0065] Rosenblatt, A. E. , & Schmitz, O. J. (2016). Climate change, nutrition, and bottom‐up and top‐down food web processes. Trends in Ecology & Evolution, 31(12), 965–975. 10.1016/j.tree.2016.09.009 27726943

[ece37273-bib-0066] Sabrina, S. , Jean‐Michel, G. , Carole, T. , Serge, B. , & Eric, B. (2009). Pulsed resources and climate‐induced variation in the reproductive traits of wild boar under high hunting pressure. Journal of Animal Ecology, 78(6), 1278–1290. 10.1111/j.1365-2656.2009.01579.x 19549145

[ece37273-bib-0067] Sandland, G. J. , & Minchella, D. J. (2003). Costs of immune defense: An enigma wrapped in an environmental cloak? Trends in Parasitology, 19(12), 571–574. 10.1016/j.pt.2003.10.006 14642767

[ece37273-bib-0068] Sheldon, B. C. , & Verhulst, S. (1996). Ecological immunology: Costly parasite defences and trade‐offs in evolutionary ecology. Trends in Ecology & Evolution, 11, 317–321. 10.1016/0169-5347(96)10039-2 21237861

[ece37273-bib-0069] Soler, J. J. , Neve, L. D. , Pérez‐Contreras, T. , Soler, M. , & Sorci, G. (2003). Trade‐off between immunocompetence and growth in magpies: An experimental study. Proceedings of the Royal Society of London. Series B: Biological Sciences, 270(1512), 241–248. 10.1098/rspb.2002.2217 12614572 PMC1691245

[ece37273-bib-0070] Sparkman, A. M. , & Palacios, M. G. (2009). A test of life‐history theories of immune defence in two ecotypes of the garter snake, *Thamnophis elegans* . Journal of Animal Ecology, 78, 1242–1248. 10.1111/j.1365-2656.2009.01587.x 19622081

[ece37273-bib-0071] Stearns, S. C. (1989). Trade‐offs in life‐history evolution. Functional Ecology, 3(3), 259–268. 10.2307/2389364

[ece37273-bib-0072] Stuart, S. N. , Chanson, J. S. , Cox, N. A. , Young, B. E. , Rodrigues, A. S. , Fischman, D. L. , & Waller, R. W. (2004). Status and trends of amphibian declines and extinctions worldwide. Science, 306(5702), 1783–1786. 10.1126/science.1103538 15486254

[ece37273-bib-0073] Sumargo, E. , & Cayan, D. R. (2018). Unusual Warmth in California's Central Sierra Nevada during the 2012‐2015 Drought. In *AGU Fall Meeting Abstracts*.

[ece37273-bib-0074] Swain, D. L. , Tsiang, M. , Haugen, M. , Singh, D. , Charland, A. , Rajaratnam, B. , & Diffenbaugh, N. S. (2014). The extraordinary California drought of 2013/2014: Character, context, and the role of climate change. Bulletin of the American Meteorological Society, 95(9), S3–S7.

[ece37273-bib-0075] Thomas, C. D. , Cameron, A. , Green, R. E. , Bakkenes, M. , Beaumont, L. J. , Collingham, Y. C. , Erasmus, B. F. N. , de Siqueira, M. F. , Grainger, A. , Hannah, L. , Hughes, L. , Huntley, B. , van Jaarsveld, A. S. , Midgley, G. F. , Miles, L. , Ortega‐Huerta, M. A. , Townsend Peterson, A. , Phillips, O. L. , & Williams, S. E. (2004). Extinction risk from climate change. Nature, 427(6970), 145–148. 10.1038/nature02121 14712274

[ece37273-bib-0076] Uhrig, E. J. , Spagnoli, S. T. , Tkach, V. V. , Kent, M. L. , & Mason, R. T. (2015). Alaria mesocercariae in the tails of red‐sided garter snakes: Evidence for parasite‐mediated caudectomy. Parasitology Research, 114(12), 4451–4461. 10.1007/s00436-015-4686-6 26337267 PMC4605875

[ece37273-bib-0077] Urban, M. C. (2015). Accelerating extinction risk from climate change. Science, 348(6234), 571–573. 10.1126/science.aaa4984 25931559

[ece37273-bib-0078] Vermeulen, A. , Müller, W. , Matson, K. D. , Tieleman, B. I. , Bervoets, L. , & Eens, M. (2015). Sources of variation in innate immunity in great tit nestlings living along a metal pollution gradient: An individual‐based approach. Science of the Total Environment, 508, 297–306. 10.1016/j.scitotenv.2014.11.095 25489975

[ece37273-bib-0079] Vose, R. S. , Applequist, S. , Squires, M. , Durre, I. , Menne, M. J. , Williams, C. N. Jr, Fenimore, C. , Gleason, K. , & Arndt, D. (2014) NOAA's Gridded Climate Divisional Dataset (CLIMDIV). California Climate Division 3. NOAA National Climatic Data Center.

[ece37273-bib-0080] Walls, S. , Barichivich, W. , & Brown, M. (2013). Drought, deluge and declines: The impact of precipitation extremes on amphibians in a changing climate. Biology, 2(1), 399–418. 10.3390/biology2010399 24832668 PMC4009861

[ece37273-bib-0081] Walther, G.‐R. , Post, E. , Convey, P. , Menzel, A. , Parmesan, C. , Beebee, T. J. C. , Fromentin, J.‐M. , Hoegh‐Guldberg, O. , & Bairlein, F. (2002). Ecological responses to recent climate change. Nature, 416(6879), 389. 10.1038/416389a 11919621

[ece37273-bib-0082] Werner, E. E. , & Anholt, B. R. (1993). Ecological consequences of the trade‐off between growth and mortality rates mediated by foraging activity. The American Naturalist, 142(2), 242–272. 10.1086/285537 19425978

[ece37273-bib-0083] Zani, P. A. , & Stein, S. J. (2018). Field and laboratory responses to drought by Common Side‐blotched Lizards (*Uta stansburiana*). Journal of Arid Environments, 154, 15–23. 10.1016/j.jaridenv.2018.03.001

